# Neuron Membrane Trafficking and Protein Kinases Involved in Autism and ADHD

**DOI:** 10.3390/ijms16023095

**Published:** 2015-01-30

**Authors:** Yasuko Kitagishi, Akari Minami, Atsuko Nakanishi, Yasunori Ogura, Satoru Matsuda

**Affiliations:** Department of Food Science and Nutrition, Nara Women’s University, Kita-Uoya Nishimachi, Nara 630-8506, Japan; E-Mails: y_kitagishi@live.jp (Y.K.); laa_minami@cc.nara-wu.ac.jp (A.M.); GAH00635@nifty.com (A.N.); pl0okm9ijn@live.jp (Y.O.)

**Keywords:** autism, attention deficit hyperactivity disorder, neurobeachin, CADM1, dopamine transporter, membrane trafficking

## Abstract

A brain-enriched multi-domain scaffolding protein, neurobeachin has been identified as a candidate gene for autism patients. Mutations in the synaptic adhesion protein cell adhesion molecule 1 (CADM1) are also associated with autism spectrum disorder, a neurodevelopmental disorder of uncertain molecular origin. Potential roles of neurobeachin and CADM1 have been suggested to a function of vesicle transport in endosomal trafficking. It seems that protein kinase B (AKT) and cyclic adenosine monophosphate (cAMP)-dependent protein kinase A (PKA) have key roles in the neuron membrane trafficking involved in the pathogenesis of autism. Attention deficit hyperactivity disorder (ADHD) is documented to dopaminergic insufficiencies, which is attributed to synaptic dysfunction of dopamine transporter (DAT). AKT is also essential for the DAT cell-surface redistribution. In the present paper, we summarize and discuss the importance of several protein kinases that regulate the membrane trafficking involved in autism and ADHD, suggesting new targets for therapeutic intervention.

## 1. Introduction

Cell polarity and vesicle sorting are important processes that influence normal cell functions such as migration, adhesion, and neurotransmission [1]. In particular, the progression of membranes of the secretory and endocytic pathway is predominantly organized. Maintenance of the endomembrane physical design needs a stability of lipid flows of the various compartments. In addition, proteins destined for various organelles must be correctly sorted. Endosomes can bud inwardly from the membranes to form the vesicles, which receive cargo from the cell surface via endocytosis and biosynthetic cargo from the late Golgi complex [2]. Spatiotemporally, the endosomal membrane trafficking is regulated, which confirms appropriate delivery of cargo via the pathway. Membrane trafficking is indispensable for a wide range of developmental processes, which also require the interplay of several proteins and lipids [3,4]. The precise coordination of membrane trafficking is controlled by protein phosphorylation. In particular, phosphatidylinositol 3,4,5 trisphosphate (PIP3) is crucial for the membrane trafficking of early endosomes [5]. Compatibly, disruption of the PIP3 synthesis by wortmannin, a Phosphoinositide 3-kinase (PI3K) inhibitor, severely affects the formation of internal vesicles and the maturation of endosomes [6]. In addition, membrane trafficking can be induced by cAMP dependent PKA activity [7]. Changes in the activity of PKA provoke a variety of effects on the intracellular membrane dynamics including membrane sorting and trafficking [8].

In neurons, the machineries for membrane trafficking must be complex. Because of the long cell extensions, neurons have to form and keep a large membrane area, which is organized into the axonal macrodomains with specific protein compositions at synaptic sites [9]. Understandably, the assembly of central nervous synapse requires the polarized targeting of numerous proteins to synaptic compartments [10]. Establishment of the asymmetric organization of cellular components, called cell polarity, involves various processes containing membrane trafficking events and cytoskeletal dynamics [11], which is implicated in morphogenesis of various cellular organisms [12]. Cell polarization is essential for a cell to function properly. For example, the presence of an axon in neuronal cells determines the directional flow of the signal. Dysregulation of the cell polarity can cause developmental disorders. In recent years, there has been accumulating genetic evidence that links the components of membrane trafficking of intracellular vesicles to a variety of neurological conditions including autism and attention deficit hyperactivity disorder (ADHD) [13,14]. Symptoms of autism and ADHD often co-occur [15]. As neurodevelopmental disorders, both autism and ADHD share some phenotypic similarities, but are characterized by distinct diagnostic criteria [15], imposing a major impediment to childhood development and a significant burden on society. A recent study provided new insight on mechanisms of the disorders and opens up new possibilities for therapeutic intervention [16]. Here, we summarize evidence for the importance of several protein kinases that regulate the membrane trafficking involved in autism and ADHD.

## 2. Relationship between Autisms and Neurobeachin

**Figure 1:**
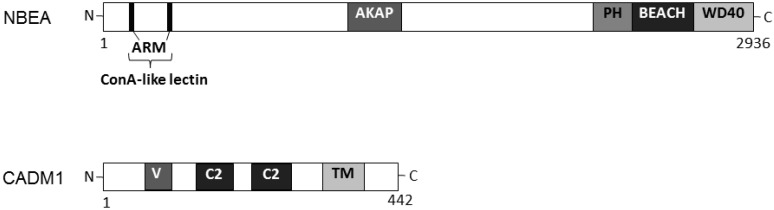
Schematic diagram representing the primary domain structures of neurobeachin (NBEA) and cell adhesion molecule 1 (CADM1) proteins. The functionally important sites are depicted. Note that the sizes of the proteins are modified for clarity. ARM, armadillo-typed domain; ConA, concanavalin A; AKAP, A-kinase anchor protein; PH, pleckstrin homology; BEACH, Beige and Chediak-Higashi domain; WD40, β-transducin repeat domain; V, variable-set Ig domain; C2, C2-set Ig domain; TM, transmembrane domain.

Autism and autism spectrum disorder (autisms) are a prevalent developmental disorder characterized by severe and sustained impairments in social interaction, repetitive restricted communication and fixed behavior. The neuropathology of autisms seems to be a defect in neurogenesis and/or its dysplastic changes in the central nervous system [17]. Although several susceptible gene loci related to autisms have been expansively studied, the molecular pathogenesis of autisms has not been well understood. The genetic structure of autisms may be heterogeneous. However, there is increasing interest in the signaling pathways associating autism-pathology with cellular functions, such as neurite outgrowth of evolving neurons and the synaptic functions [18]. So far, it has been found that many genes that are mutated, disrupted, and/or deleted in autism patients [19] may be involved in certain function and signal transduction of autisms-related cellular biology. Autism patients with a monoallelic deletion of the gene have also been reported [20]. Among the candidate genes, the *neurobeachin* gene was identified in a patient with a genetic disorder [20,21]. A single nucleotide polymorphism (SNP) of *neurobeachin* gene has also been found to associate with autisms [21]. The *neurobeachin* gene encodes a multidomain neuron-specific protein that is principally expressed in brain [22,23]. The protein is a member of the BEACH protein family implicated in membrane trafficking [22,23], in which the BEACH domain is headed by an unusual pleckstrin homology (PH) domain, and followed by a tryptophan-aspartic acid repeat (WD40) repeat domain [24] (Figure 1). The neurobeachin protein may be a negative regulator of notch function associated with the synaptic plasma membrane and involved in endosomal trafficking [25]. In addition, a function for neurobeachin in altering the actin cytoskeleton has been suggested [26]. This scaffolding protein has been suggested to be involved in neuronal *trans*-Golgi membrane traffic [20,22]. Actually, neurobeachin in *Drosophila* has been linked to the membrane trafficking of growth factor receptors [27]. Neurobeachin concentrates near the *trans*-Golgi network, suggesting a functional association to the post-Golgi sorting of membrane trafficking proteins [22]. High expression of neurobeachin seems to be limited to neuronal cells and endocrine cells [28]. It has been shown that knockout of the *neurobeachin* gene in two independent mouse models prevents an activity in synaptic function with neurotransmitter release [20]. Similarly, knockdown of neurobeachin in a neuroendocrine cell line (βTC3 cells) has shown a role as negative regulator of secretion of vesicles [20]. Insufficiency of the neurobeachin function results in dense granules with an aberrant morphology [20]. New understandings in the function of neurobeachin may support identifying novel molecular pathways affected in neurons with autistic patients [29,30,31].

## 3. Relationship between Autisms and Cell Adhesion Molecule 1 (CADM1)

Although mutations in genes encoding neurobeachin have been shown in autism patients, the consistent idea on the molecular pathogenesis of autisms is still unknown. Cell-adhesion molecule 1 (CADM1, TSLC1/SynCAM1) is a member of the immunoglobulin (Ig) superfamily containing extracellular Ig-like loops, a single transmembrane domain, and a small intracellular carboxy-terminal tail, is another synaptic cell adhesion molecule [32,33] (Figure 1). CADM1 mRNA is expressed diffusely in the lateral membrane of cell-cell attachment sites in polarized epithelia, and is also expressed on rod photoreceptors in a developmentally regulated manner [32,33]. In addition, the CADM1 is expressed not only in various regions of the cerebrum but also in the developing cerebellum [34,35,36]. Mutations in CADM1 are associated with autisms [34,35,36]. The mutated CADM1 shows morphological abnormalities including impaired synaptogenesis in mice model neurons [35]. CADM1 co-localizes with alpha-bungarotoxin at the neuromuscular junctions and interacts with the multiple PDZ domain protein Mupp1, a scaffold protein containing PDZ domains [37]. In addition, CADM1 localizes on the dendrites in molecular layers of developing cerebellum as well as on the dendrites of hippocampal neurons [35]. Accordingly, CADM1 synaptic receptor complex may be associated with autisms pathogenesis locating on the dendrites of neuron cells. Cerebellar aberrations including Purkinje cell damage have been shown in autisms patients [38]. Furthermore, the autism-related mutations of CADM1 may bring defective membrane trafficking at the mouse neuronal cell surface [39], suggesting that a link between impaired synaptogenesis and the molecular pathogenesis of autisms [39]. In fact, the CADM1-knock out mice exhibit small cerebellums with decreased numbers of synapses with Purkinje neuron cells, which show some similar behaviors associated with autisms [36]. The mutated CADM1 also exhibits defective membrane trafficking and greater susceptibility to the cleavage and/or degradation [39], which is essential for trans-active molecular interaction [39]. In addition, CADM1 is localized to the thalamus cortical afferent pathway in the cerebrum. Mutations in CADM1 may increase its susceptibility to processing errors and the accumulation of some CADM1 degradation products in the endoplasmic reticulum [40], which may diminish CADM1 function in cell adhesion and result in synaptic disorders in neurons. Impaired synaptogenesis then underlies the pathogenesis of autisms. Actually, CADM1 has homo-dimer aggregation activity when introduced into Madin-Darby canine kidney cells (MDCK) cells lacking endogenous CADM1 expression in a Ca^2+^/Mg^2+^ independent manner [41], indicating that CADM1 is involved in cell adhesion through homophilic trans-interaction [41]. However, the cytoplasmic signaling pathways started by CADM1 have not been fully elucidated. Epigenetic factors may also complicate the understanding of pathogenesis in autisms. The example of exposure to valproate provides a good illustration of epigenetic mechanisms involved in autisms [42].

## 4. Relationship between Attention Deficit/Hyperactivity Disorder (ADHD) and Dopamine Transporter (DAT)

**Figure 2:**
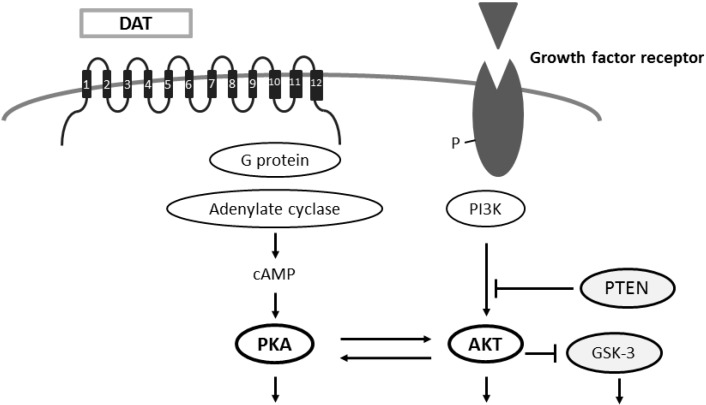
Schematic illustration of intracellular dopamine transporter (DAT) signaling with PKA and AKT pathways has been shown. Arrowhead means stimulation whereas hammerhead represents inhibition. Note that some critical pathways have been omitted for clarity.

Attention deficit/hyperactivity disorder (ADHD) is associated to dopaminergic insufficiencies in prefrontal cortex [43], which is a heterogeneous disorder typically diagnosed in school-age children. ADHD is characterized by hyperactivity, impulsivity, and inappropriate levels of inattention. Although studies suggest a contribution of altered dopamine signaling in ADHD brains, evidences of signaling disturbances contributing to the risk of ADHD may be often conditional [14,44]. On the other hand, some studies have pointed to a contribution of variation in the genes encoding dopamine transporter (DAT), dopamine receptors, and/or catechol-*O*-methyl transferase (COMT) as influencing risk for ADHD [45,46]. Remarkably, all of these are involved in signal transduction at the neuronal synapse. In particular, a link between DAT function and ADHD is mostly suggested from the therapeutic utility of DAT-interacting psychostimulants such as amphetamine (AMPH) and methylphenidate. ADHD is attributed to dysfunction of DAT in the prefrontal cortex [47]. Removal of DAT expression in animal models decrease presynaptic dopamine stores [48] and produces hyperactivity [49,50]. Altered DAT expression also affects age-related changes in dopaminergic function [50]. ADHD is associated with increased DAT expression in striatum [49,50], and with specific polymorphisms in the *DAT* gene [46,51]. Furthermore, methylphenidate is a standard successful treatment for ADHD, which is an inhibitor of DAT and norepinephrine transporters [52]. In a well-established animal model of ADHD, spontaneously hypertensive rat (SHR), methylphenidate recovers the abnormal behaviors including attention-deficit and hyperactivity to a certain extent [53]. Methylphenidate may act as an inhibitor of striatal and prefrontal cortical DAT function, increasing extracellular dopamine concentrations [54,55]. Dopamine receptor-blockade could be considered as a novel treatment approach for symptoms observed in ADHD [54]. The presynaptic AMPH-sensitive DAT restrains dopamine availability at the synaptic receptors following vesicular release [14]. While mutants of DAT proteins show insensitivity to the endocytic effects of AMPH, phosphorylation of DAT may be involved in sorting DAT in regulated pathways [14]. Re-uptake of dopamine through presynaptic DAT is a chief mechanism for dismissing dopamine action at synaptic receptors. DAT is also a molecular target for therapeutic treatment used in mental disorders such as depression and Parkinson’s disease [56]. The DAT-mediated re-uptake system controls the intensity as well as the duration of dopamine actions at synaptic receptors, which provides critical modulatory influences over attention and behaviors [57]. Therefore, dopamine signaling is a crucial risk factor for ADHD. DAT may be critically involved in the dopaminergic dysfunction associated with ADHD. Importantly, the intra-cellular signaling of DAT may go through the PKA and AKT pathways (Figure 2).

## 5. Neuronal Membrane Trafficking Involved in Autisms and ADHD Regulated by Several Protein Kinases

The amino-terminal region of neurobeachin with a concanavalin A-like domain fringed by armadillo repeats may play an important role in intracellular membrane trafficking. Distal from these regions, an A-kinase anchoring protein (AKAP) domain functions to recruit cAMP-dependent protein kinase A (PKA) by binding to its regulatory RIIα subunit (Figure 1). PKA is a collective term for an enzyme family containing three catalytic subunit isoforms and four regulatory subunit isoforms. Neurobeachin belongs to the AKAP family of proteins, which is known to scaffold PKA near its target proteins in a subcellular compartment. In neurobeachin haplo-insufficiency mice, the level of brain-derived neurotrophic factor (BDNF) is increased, which is one of the targets of cAMP response element-binding protein (CREB) transcription [58]. Similarly, after knockdown of the neurobeachin expression, PKA-mediated phosphorylation of CREB is increased in a neuronal cell line. The modified PKA phosphorylation of different proteins affected by neurobeachin could be explained by the effect on AKAP function, an altered PKA-mediated phosphorylation of target proteins depending on its subcellular localization [59]. Neurobeachin appears to be important for the formation and composition of central synapses [59]. There is genetic evidence for the involvement of AKAP function to integrate signaling cascades in the etiology of autisms [26]. Pleiotropic effects of alterations in PKA activity due to neurobeachin were demonstrated, with an important function of the AKAP domain limiting PKA activity [26], suggesting a role for neurobeachin in remodeling the actin cytoskeleton [26]. PKA is regulated through the cAMP second messenger in response to a variety of extracellular signals, following activation of different intracellular pathways including that of membrane trafficking [60]. Regulation of PKA action is thought to be facilitated in part by AKAPs [61], which cause an increase in cAMP, then activates catalytic subunits of the PKA inactive enzymes. AKAPs are linked to synaptic sites and microfilaments [62,63], which are implicated in the PKA-regulation of certain physiological synaptic events, including modulation of neurotransmitter receptors and the exocytosis of synaptic vesicles. For example, dendritic spine formation requires neurobeachin [64]. As actin is a spine-associated protein, a role emerges for neurobeachin in trafficking cargo to synaptic compartments [64].

CADM1 is also linked to the actin cytoskeleton [65]. In addition, it has been shown that CADM1 associates with members of a group of scaffolding proteins and/or membrane-associated guanylate kinase homologs [66]. Several membrane-associated guanylate kinase homologs are localized at the synaptic regions, working on a synaptic plasticity through the clustering of receptors [67]. Cytoplasmic CADM1domain recruits PI3K to the juxta-membrane region in order to induce actin reorganization by activating AKT, which then results in cell spreading [65]. When CADM1 is activated, the AKT may be a key molecule downstream of the signaling [65,68]. Consistently, some of PI3K and AKT inhibitors show an activity of inhibiting the cell spreading [68]. So, the PI3K/AKT pathway seems to be important for the signals mediated by CADM1, which may also represent a novel mechanism for regulating dopamine efflux induced by AMPH through DAT modulation [69,70]. AKT is essential for the DAT cell-surface redistribution [69]. Likewise, insulin regulates dopamine clearance through the PI3K/AKT signaling by DAT membrane expression [71]. Several PI3K/AKT kinase modulators may exert principal effects on DAT cellular distribution [72]. In addition, inhibition of PI3K decreases the DAT on cell surface expression [73]. High dopamine concentrations reduce uptake velocities in the presence of LY294002, a well-known PI3K/AKT inhibitor, suggesting that PI3K/AKT mediates substantial effects on DAT function [74]. Synaptic dopamine signaling may also be altered through a reduction of the available cell surface DAT via the modulation of PI3K/AKT activity.

## 6. Interplay of the Kinases Involved in Autisms and ADHD

**Figure 3:**
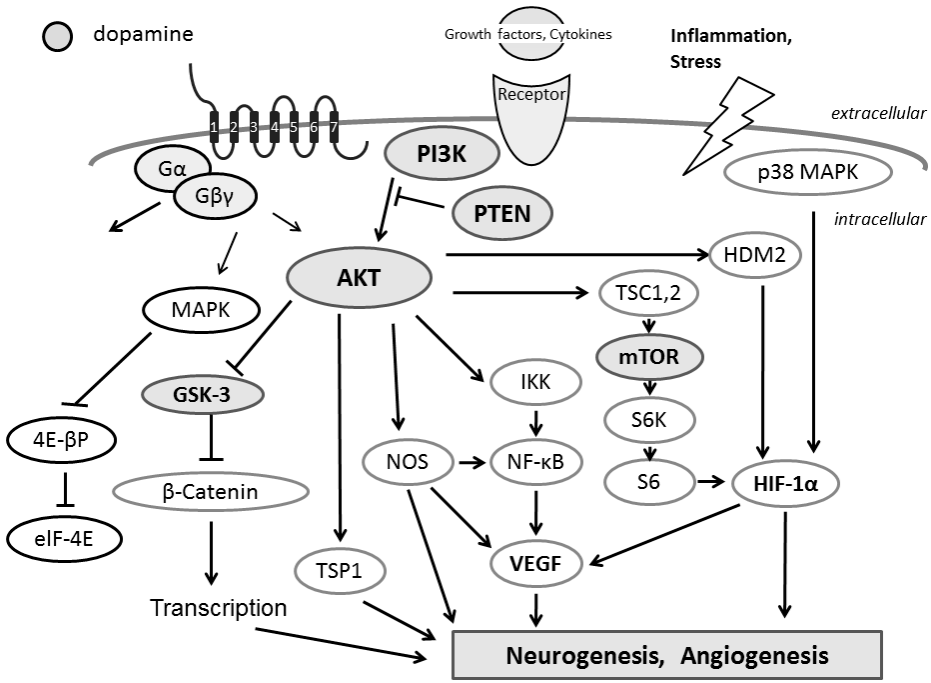
Schematic depiction and overview of PI3K/AKT/PTEN signaling has been shown. Example molecules known to act on the PI3K/AKT/PTEN pathway are also shown. Arrowhead means stimulation whereas hammerhead represents inhibition. Note that some critical pathways have been omitted for clarity.

AKT is a central player in signal transduction activated in response to several growth factors, which is thought to contribute many important cellular functions, including cell growth, apoptosis, nutrient metabolism, and modulating the activity of various transcription factors [75] (Figure 3). AKT is subjected to phosphorylation-regulation by phosphoinositide-dependent kinase 1 (PDK1) at Thr308. Full activation of AKT requires further phosphorylation of its Ser473 at the carboxyl-terminus by kinases such as PDK2 and the mTOR complex 2 (mTORC2) [76]. The PKA signaling is involved in affecting the GSK-3β phosphorylation status at phospho-GSK-3β (Ser9) [77], which is also a downstream target of PI3K/AKT signaling. GSK-3 contains α and β isoforms, which is an important kinase involved in the regulation of a group of transcription factors [78]. Evidence suggests that lithium causes its neuro-protective effects predominantly through inhibition of the GSK-3 [79]. Similarly, the development of GSK-3 isoform-specific inhibitors seems to be warranted for treating GSK-3-mediated pathology [79]. Lithium may also modify GSK-3 activity through phosphorylation both of GSK-3α and GSK-3β by various mechanisms including the activation of PKA and AKT [80,81], indicating that lithium exerts its potentiating and inhibiting bidirectional cellular actions on GSK-3 activity. In addition, inhibition of GSK-3 seems to be involved in the antagonistic effects of lithium on depressant and manic properties [82]. In mouse photoreceptor-derived 661W cells, bFGF signaling inactivates GSK-3β by phosphorylation at Ser9, which is dependent on PKA activation [83]. A pharmacological inhibitor of PKA can antagonize the GSK-3β Ser9 phosphorylation [84], supporting its potential use in chemotherapeutic options.

PKA and AKT have been shown to establish complexes with AKAP150, which may act as a key regulator to control AKT phosphorylation. PKA activation leads to a reduction of AKT phosphorylation. In diverse neuronal processes ranging from neuronal survival to synaptic plasticity, cAMP-dependent signaling is tightly connected with the AKT signaling pathway [85]. In addition, a crosstalk between PKA and mammalian target of rapamycin (mTOR) pathway in apoptosis resistance signaling has been reported [86]. mTOR, a conserved serine/threonine kinase within cells, is a key molecule in controlling protein synthesis and cell growth, and also involved in neurological disorders including autisms and ADHD [87]. To achieve a better quality of life for those patients, therapy approaches are directed at restoring dysregulated mTOR signaling [87]. mTOR complex 1 (mTORC1) and mTOR complex 2 (mTORC2) are two distinct complexes with mTOR, which are also involved in autisms and ADHD [88]. Loss of mTORC2 signaling in the cortex independent of mTORC1 might disrupt normal brain development and its function [88]. The mTORC1 pathway is activated by the PKA signaling, leading to increased cell survival, which is correlated with BAD hyper-phosphorylation. Furthermore, PKA and mTOR signaling cascades are important even for the development of follicular thyroid carcinogenesis, suggesting new targets for therapeutic intervention [89].

## 7. Diets May Contribute to the Improved Membrane Trafficking in Autisms and ADHD via the Modulation of AKT and PKA Signaling

Dietary depletion of tryptophan, which is the precursor of serotonin, has been shown to exacerbate repetitive behavior in autistic patients [90]. Tryptophan-restricted animals show a reduced activity of phosphorylated AKT [91]. In addition, serotonin promotes interactions with a scaffolding and regulatory protein, which results in the activation of AKT [92]. Consistently, studies have found elevated serotonin levels in whole blood cells and platelets of autism patients [93,94] and changes in serotonin transporter function have been implicated in autisms [95]. Enhanced activity of serotonin transporter variants is also associated with autisms [96]. Curcumin, a component in the widely used culinary spice turmeric, can improve structure and plasticity of synapse and enhance memory abilities [97]. It is suggested that the neuroprotection of curcumin might be mediated via PI3K/AKT signaling pathway [98,99]. In the function of membrane trafficking, curcumin seems to be able to restore a functional cystic fibrosis transmembrane conductance regulator (CFTR) to the plasma membrane [100]. Furthermore, curcumin and genistein additively potentiate the function of the CFTR [101]. Curcumin potently decreases amyloid-β levels in the secretory pathway [102]. Furthermore, an obvious anxiolytic effect of curcumin has been shown in a lead-induced anxiety animal model, possibly resulted from modulation of central neuronal serotonin neurotransmission [103]. Recently, omega-3 (ω-3) long-chain polyunsaturated fatty acids (PUFAs) have become a focus of interest. Especially, docosahexaenonic acids (DHA) are essential for brain development and physical health. The symptoms of ADHD have been suggested to be associated with a deficiency of the ω*-3* PUFAs [104]. In addition, low blood ω*-3* PUFAs have been reported in children with ADHD and related learning difficulties, suggesting benefits from dietary supplementation [105]. Fish oil administration was reported to protect hippocampal neurons and improves cognitive deficit by increasing AKT phosphorylation [106]. In addition, neuroprotecton could be performed by certain diets involved in the PI3K/AKT pathway [107,108]. Several fruits may be promising. Kaempferol is a flavonol that is present in various plants including grapefruit and some edible berries, which also induces the activation of PI3K and AKT [109]. On the contrary, the biological activity of the isothiocyanates, rich in certain vegetables such as broccoli, has been shown to suppress AKT phosphorylation [110]. However, despite these experimental observations, the precise mechanisms for these food ingredients remain elusive further for the clinical uses. Additionally, it seems important to exploit the potential benefits of optimal treatment and/or combination with these PI3K/AKT modulators.

Overexpression of phosphatase and tensin homologue deleted on chromosome 10 (PTEN) has been shown to have inhibitory effects on serotonin signaling via decreased AKT activity [111]. PTEN negatively regulates activity of the PI3K/AKT pathway, which is a dual-specificity phosphatase acting as both protein phosphatase and lipid phosphatase that suppresses PI3K activity through converting PIP3 to PIP2 [112]. Honokiol, a chemical compound in traditional eastern herbal medicines, can attenuate the PI3K/AKT signaling by up-regulation of PTEN expression [113]. Dietary and/or therapeutic interventions to counteract the reduction of PTEN expression could contribute to the prevention of the diseases and/or decrease the rate of its development. Accordingly, the culinary herb sage (*Salvia officinalis*) may be unhelpful for autism patients [114]. However, PTEN indirectly promotes serotonin synthesis and secretion via inhibiting the signaling [115]. In addition, there is a crosstalk between PTEN and the serotonin receptor [116]. It has been shown that docosahexaenonic acids (DHA) and eicosapentaenoic acid (EPA) increase the level of PTEN in breast cancer cells, providing a mechanism for the beneficial effects of fish oils even on cancer cell growth [117,118]. Since DHA and EPA are ligands of PPARγ, both of the ω-3 PUFAs exert anti-proliferative effects by inducing PTEN via the activation of the PPARγ [119]. Controversially, phosphorylated AKT may be down-regulated by treatment with curcumin due to the activation of PTEN. In addition, the most attractive target for phytoestrogen with regard to PTEN transcription seems to be PPARγ [120]. Both genistein and quercetin also have an effect on PPARγ activation, which has been shown to up-regulate PTEN transcription, then, suppresses the PI3K/AKT pathway [121]. Dietary exposure to the soy isoflavones at physiologically relevant concentrations induces PTEN expression [122]. Generally, phytoestrogen exposure may result in an increase in PTEN expression. In addition, a high-fat diet raises circulating fatty acids, which significantly alters PTEN expression [123]. Interestingly, rosemary extract was reported to repress PTEN expression in K562 leukemic culture cells [124]. Again, indole-3-carbinol is a promising cancer-preventive phytochemical found in some vegetables such as broccoli. Dietary intake of the indole-3-carbinol up-regulates PTEN in the mouse model [125].

**Figure 4:**
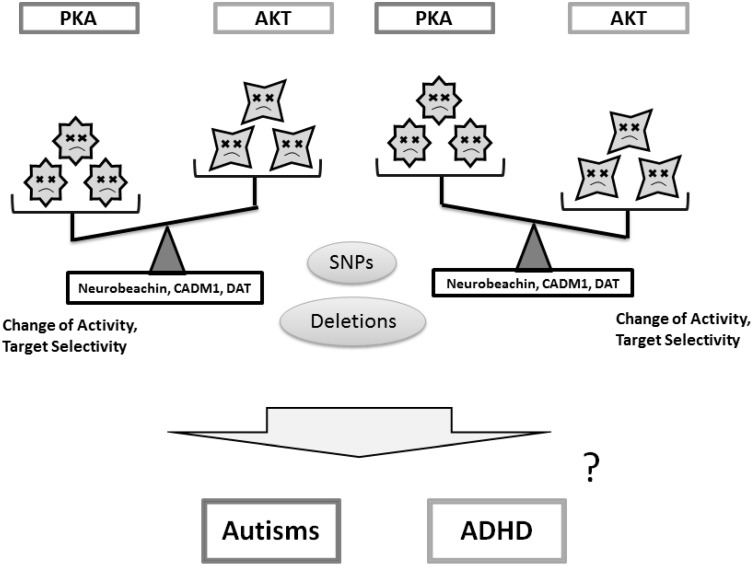
Schematic implication of protein kinases PKA and AKT modulation in the pathogenesis of autisms and ADHD. Alteration of the functions in neurobeachin, CADM1 and DAT with genetic deletion and/or single nucleotide polymorphisms (SNPs) may change the activity or selectivity of kinase to substrates, which in turn may cause the psychological disorders. Star faces represent an image of the individual kinase activities. Sad faces mean unbalance of kinase activity.

**Figure 5:**
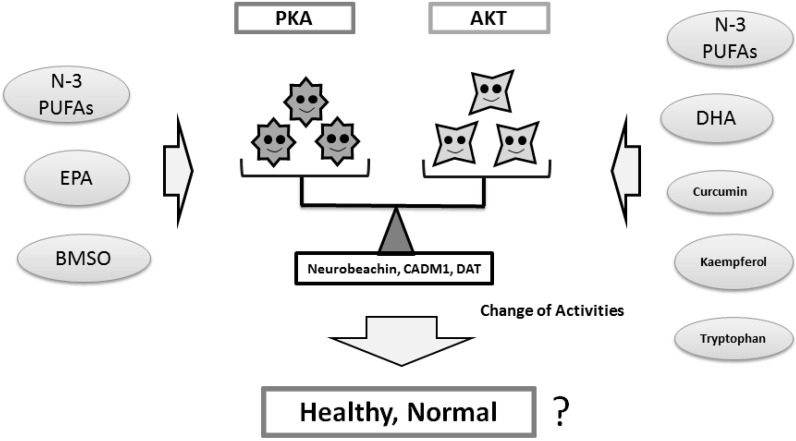
The balance of PKA and AKT kinases in the meaning of their functions may be important for normal neuronal development and individual psychiatric health. Several food and/or dietary components may contribute to the improved balance of the AKT and PKA signaling via the modulation of kinase activities. Star faces represent an image of the individual kinase activities. Smiled faces mean appropriate balance of kinase activity.

The PKA pathway regulates cell growth and division in response to nutrient status [126]. PKA is activated by the ω-3 PUFAs EPA [127]. Bitter melon seed oil (BMSO), which is rich in the isomers of conjugated linolenic acid, increases phosphorylation and activation of PKA [128]. In addition, genistein directly activates the cAMP/PKA cascade [129]. Very low protein diets result in a desensitization of cAMP signaling, which is characterized by a loss of PKA activity [130], suggesting that dietary protein and energy restriction may modulate PKA activity. It seems that both activation and inhibition of those kinases, if they work one-sidedly, may not contribute to the improvement of the neuronal disorders (Figure 4). It looks that back and forth activation and/or inhibition may be important. In other words, the balance of PKA and AKT kinases may be essential for their functions. Several food and/or dietary components may contribute to the balance via the modulation of kinase activities (Figure 5). These findings might be translated into new dietary managements for the treatment of autisms and ADHD in the future.

## 8. Perspective

Occasionally, neurodegenerative diseases also exhibit defective endsomal trafficking. The vesicle transport may be necessary to ensure neurotic organelle homeostasis. Functional significance of membrane trafficking in the brain neuron signaling pathways remains to be more established. Roles of neural networks are defined by synapses, which are adhesions specialized for intercellular communication. Neurobeachin, CDM1 and DAT may act as crucial regulators in the synaptic neuron for the harmonization of morphology with synaptogenesis. Neuronal activity triggers gene transcription, thereby regulating synaptic function. Many of the genes that are mutated in autisms are key components of the activity-dependent neural networks that regulate synapse development and plasticity [131]. Therefore, dysregulation of activity-dependent signaling pathways in neurons may have a key role in the etiology of autisms. In particular, phosphorylation has appeared as a critical regulatory mechanism in neurons to incorporate the dynamic signaling required for appropriate synaptic function, development, and plasticity. As mentioned above, it has been suggested that supplementation to correct low levels of DHA and as well as low levels of other ω*-3* PUFAs are predicted to improve learning and behavior problems in healthy children. Accordingly, one treatment model is based on the reduced levels of the ω*-3* PUFAs in ADHD children [132]. The benefits from dietary supplementation of ω*-3* PUFAs may extend to a wider population. There is now substantial evidence that ω*-3* PUFAs deficiency is associated with patho-physiological mechanisms implicated in the progression of different psychiatric disorders. In addition, emerging evidence suggests that ω*-3* PUFAs augment the therapeutic efficacy of antidepressants and mood-stabilizers [133]. Importantly, the ω*-3* PUFAs may be involved in membrane trafficking via the modulation of PI3K/AKT activity. Uptake of the PUFAs into endo-membranes may alter the rate of trafficking of molecules [134]. Membrane modification of neuronal development appears to be an important mechanism of the antipsychotic effects by the PUFAs. In addition, the ω*-3* PUFAs have an established long-term safety, and the total cost-benefit ratio provides a validation for the psychiatric treatment-protocols. Deciphering the precise mechanisms of the pathology will offer new insight into the physiological roles in regulating membrane trafficking. It is expected that future studies will address this area to gain a better understanding of the potential and specific signaling molecules involved in autisms and ADHD.

## Abbreviation

ADHD: Attention deficit/hyperactivity disorder
AMPH: Amphetamine
AKAPs: A-kinase anchor proteins
AKT: Protein kinase B
BDNF: Brain-derived neurotrophic factor
bFGF: Basic fibroblast growth factor
BEACH: BEige and Chediak-Higashi
BMSO: Bitter melon seed oil
CADM1: Cell adhesion molecule 1
CFTR: Functional cystic fibrosis transmembrane conductance regulator
cAMP: Cyclic adenosine monophosphate
COMT: Catechol-*O*-methyl transferase
CREB: cAMP response element-binding protein
DAT: Dopamine transporter
DHA: Docosahexaenonic acids
EPA: Eicosapentaenoic acid
GSK-3: Glycogen synthase kinase 3
Ig: Immunoglobulin
mTOR: Mammalian target of rapamycin
mTORC1: mTOR complex 1
mTORC2: mTOR complex 2
NBEA: Neurobeachin
PDK1: Phosphoinositide-dependent kinase 1
PDK2: Phosphoinositide-dependent kinase 2
PDZ: PSD-95/Dlg/ZO-1
PH: plekstrin homology
PIP3: Phosphatidylinositol 3,4,5-triphosphate
PI3K: Phosphatidylinositol-3 kinase
PKA: Protein kinase A
PPARγ: Peroxisome proliferator-activated receptor
PTEN: Phosphatase and tensin homologue deleted on chromosome 10
PUFAs: Polyunsaturated fatty acids
SHR: Spontaneously hypertensive rat
SNP: Single nucleotide polymorphism
WD40: tryptophan-aspartic acid repeat
